# New directions in biomedical text annotation: definitions, guidelines and corpus construction

**DOI:** 10.1186/1471-2105-7-356

**Published:** 2006-07-25

**Authors:** W John Wilbur, Andrey Rzhetsky, Hagit Shatkay

**Affiliations:** 1 National Center for Biotechnology Information NLM, NIH, Bethesda, MD, USA; 2 Department of Biomedical Informatics, Center for Computational Biology and Bioinformatics, Judith P. Sulzberger MD Columbia Genome Center, and Department of Biology, Columbia University, New York, NY, USA; 3 School of Computing, Queen's University, Kingston, ON, Canada

## Abstract

**Background:**

While biomedical text mining is emerging as an important research area, practical results have proven difficult to achieve. We believe that an important first step towards more accurate text-mining lies in the ability to identify and characterize text that satisfies various types of information needs. We report here the results of our inquiry into properties of scientific text that have sufficient generality to transcend the confines of a narrow subject area, while supporting practical mining of text for factual information. Our ultimate goal is to annotate a significant corpus of biomedical text and train machine learning methods to automatically categorize such text along certain dimensions that we have defined.

**Results:**

We have identified five qualitative dimensions that we believe characterize a broad range of scientific sentences, and are therefore useful for supporting a general approach to text-mining: focus, polarity, certainty, evidence, and directionality. We define these dimensions and describe the guidelines we have developed for annotating text with regard to them.

To examine the effectiveness of the guidelines, twelve annotators independently annotated the same set of 101 sentences that were randomly selected from current biomedical periodicals. Analysis of these annotations shows 70–80% inter-annotator agreement, suggesting that our guidelines indeed present a well-defined, executable and reproducible task.

**Conclusion:**

We present our guidelines defining a text annotation task, along with annotation results from multiple independently produced annotations, demonstrating the feasibility of the task. The annotation of a very large corpus of documents along these guidelines is currently ongoing. These annotations form the basis for the categorization of text along multiple dimensions, to support viable text mining for experimental results, methodology statements, and other forms of information. We are currently developing machine learning methods, to be trained and tested on the annotated corpus, that would allow for the automatic categorization of biomedical text along the general dimensions that we have presented. The guidelines in full detail, along with annotated examples, are publicly available.

## Background

The past few years have seen an impressive growth in the amount of research dedicated to biomedical text mining, (see several recent reviews [1-11] and a recent edited collection [12]). The field that originally focused on medical text [13-16] has expanded since the onset of the genomic era into the biological domain. Research in the area includes work on information extraction from the biomedical literature [17-22], as well as on information retrieval and text categorization [1,2,23-26]. The efforts on information extraction mainly concentrate on identifying bio-entities (mostly genes and proteins) and the relationships among them, while current efforts on information retrieval, with a few exceptions, aim at identifying documents for specific database curation tasks and categorization of papers into various ontological types [25]. We believe that an important first step towards more accurate information extraction and retrieval, lies in the ability to identify and characterize text that satisfies certain kinds of information needs. One goal of the work presented here is to identify properties of scientific text that have sufficient generality to transcend the confines of a narrow subject area, while supporting practical mining of text for factual information.

One of the most challenging aspects of biological text-mining is deciding how to use scarce resources to annotate text as a training corpus for machine learning. There are two objectives to be considered in this context. On the one hand, the corpus should be widely useful to the biomedical data-mining research community. On the other hand, it should lead to the development of practical and useful resources. Regarding the first objective, Cohen *et al*. [27] have studied the most prominent corpora designed to promote biomedical text mining. They suggest four main characteristics of a corpus that make it user-friendly and promote a high level of use: documentation, balanced representation, recoverability, and data on inter-annotator agreement. Documentation in the form of published annotation guidelines is important [27]. One of the purposes of this paper is to address this need. Recoverability is the requirement that the original text remains available to users of the corpus. This ensures that no aspect of the original data is lost. Balanced representation requires coverage of a broad range of text types that are encountered in practice. Cohen *et al*. also suggest that abstracts may no longer be acceptable as the sole source of data. Accordingly, in the work introduced here, the majority of our data is (and will be) sampled from the different sections of full-text journal articles (A minority will still come from a random sampling of MEDLINE^® ^abstracts). With respect to inter-annotator agreement, for the full corpus, all sentences are being annotated by at least three independent annotators, and we study and monitor the agreement among them. The preliminary data we report here was annotated by twelve independent individuals, and agreement among them is the topic of much of this paper.

The second objective is to make a difference on the practical side. Our aim is to create a training corpus for automated classifiers, ultimately performing text-mining tasks that could support and expedite biomedical research. The basic task that we are aiming to address is the finding of reliable information. The fact that a gene is mentioned in the text and the text states, for example, that the gene is regulated by another gene, does not necessarily imply that the information is reliable or useful. Krauthammer et al. [28] suggested a critical examination of literature contents in molecular biology, and recent work by Light et al. [29] also examined the category of speculations versus definite statements made in the literature. As an example, one may compare the following statements taken from actual research reports:

"*We suppose that an increased LI in breast tissues of this group of patients may help explain the association between BC and thyroid autoimmunity.*" and

"*Hyphae-specific genes, HWP1, RBT4 and ECE1, were activated in the elongated filaments caused by the Cdc28p depletion.*"

The first sentence only speculates about a possible explanation for association between breast cancer and thyroid autoimmunity, but provides no evidence that it is true. The second statement, on the other hand, makes a definite assertion about the activation of genes and the relations among them. Moreover, the use of the past tense form "were" provides indication that this was an actual finding in the study being reported. One of the important observations demonstrated by this example is that no deep knowledge of the fields involved, or even of the jargon used in these fields is required to draw such conclusions. Following this line of reasoning, we devised criteria for characterizing statements made in the literature along several dimensions, based on certain types of meta-knowledge. These dimensions, which we introduce and describe in the Methods section, include focus (e.g. scientific vs. general), polarity (positive vs. negative statement), level of certainty, strength of evidence, and direction/trend (increase or decrease in certain measurement). The ultimate utility of a text-region, as a source for a certain type of scientific knowledge, can be evaluated based on its "coordinates" along these dimensions.

Prior work on annotation of scientific text (e.g. [30-33]) focused on the partition of text into zones, according to the type of discourse and the components of scientific argumentation (e.g. background, framework, aim). Teufel et al. [33] designed an annotation scheme for text involving seven rhetorical roles, such as: Background, Basis, Aim, Own, etc., borrowed from rhetorical structure theory [34]. A more extensive hierarchical tree-structured scheme, was developed by Langer et al. [30]. It consists of higher level nodes of general text types, such as Background, and Evidence, and sixteen more specific leaves such as Research Topic, Data, Results, and Conclusions. McKnight and Srinivasan [31] studied structural categories: Introduction, Method, Result, and Conclusion, which commonly appear in scientific text. All of these approaches seek to categorize scientific text, in order to improve the understanding of content, with possible application to text-mining. However, these methods differ from our approach, as they all strongly rely on predefined structural roles or types of discourse.

Among previous studies, perhaps closest to our intent is that of Mizuta and Collier [32,35,36] on Zone Analysis, where zones are based on types of discourse. Their work is based on, but significantly extends, the original framework proposed by Teufel et al. [33]. They propose seven top level classes: Background, Problem setting, Outline, Textual, Own, Connection, and Difference. The Own category is divided into five subclasses: Method, Result, Insight, Implication, and ELSE (anything else). Annotation is typically assigned to a sentence or a group of sentences, but for a specified list of clause and phrase types a lower-level of annotation has proven necessary. Due to language-complexities two levels of nested annotations are also supported.

Our present study, much like the work of Mizuta and Collier, is motivated by the need to identify and characterize locations in published papers where reliable scientific facts can be found. However, our work differs from theirs in two main aspects, namely, the complexity and the specificity of the annotation task addressed. First, the annotation scheme suggested by Mizuta and Collier is quite complex; we believe that such an intricate scheme makes both annotation and utilization of the corpus more difficult, and requires more effort to yield practically satisfying results. Second, their annotation scheme, (like its predecessors [30-33]), assumes that specific zones or types of discourse bear certain types of information. This annotation scheme ultimately limits the type of discourse and the areas in the document which can be identified, under the assumption that specific types of discourse or zones typically bear more relevant information than others. In contrast, we define a set of five general dimensions, along which each sentence or sentence fragment within the text is to be characterized – regardless of its semantic contents or zone.

We ultimately plan to develop a battery of machine-learning methods to carry out such annotation. To enable that, a set of guidelines must be crafted and a corpus of training data produced of sufficient quality to support the proposed machine learning. Our belief is that the greatest return will accrue from a relatively simple approach which envisions no sophisticated language understanding or discourse analysis. Rather, we see the task as decomposition of meta-information about text into multiple relatively independent dimensions along which a human can discern a level of organization or information, roughly at the level of sentiment analysis [37-40]. We believe this kind of data will support a sufficiently high level of machine-learning to pay practical dividends.

The rest of the paper describes aspects of the guidelines that we have developed to characterize text fragments along the multiple dimensions mentioned above. The annotation guidelines themselves are the subject of the Methods section, presented at the end of the paper. The Results section reports the results from a test we conducted to evaluate these guidelines by measuring inter-annotator agreement within two groups of annotators. It is followed by a discussion and conclusion.

## Results

### Annotation task

We have developed the guidelines over a period of more than a year, through multiple iterations of testing and revisions. Once the guidelines reached their current form, (as described in the Methods section) we designed a formal preliminary test, before proceeding to the full-corpus annotation (which is currently ongoing). Ten research articles were randomly chosen from those published in 2005, and from these articles 101 sentences were chosen at random from the different sections of the papers, at a rate of approximately ten sentences per paper. These 101 sentences form the experimental corpus for a small annotation test.

### Annotator characteristics

The three of us each annotated the test corpus independently, forming one group of annotators. Nine other independent individuals with basic scientific training (graduate students in science disciplines) also annotated the corpus, forming the second group of annotators. While we as authors have had extensive experience with the guidelines, the other nine annotators were simply given the guidelines along with appendices containing annotated examples, and were asked to read them and apply the guidelines to the corpus.

We have analyzed the resulting annotations in several ways. Table 1 summarizes the distribution of the tags assigned to the different fragments by the annotators. Its first 5 rows show the distribution of the number of fragments into which the sentences were broken by the annotators. Because there are some marked differences in the number of fragments produced per sentence, for subsequent rows we normalized the counted data, dividing counts by the number of fragments from which these counts were produced. This removes the number of fragments from affecting the comparison of the annotations along the other dimensions. There are still significant differences in annotator performance, as reflected by Table 1.

### Inter-annotator agreement

The variation in the data is not surprising. To examine the reliability of the annotations we directly examine agreement levels among annotators in several different ways, as described below. We chose not to use the familiar Kappa statistic for two reasons. First, Kappa values are not comparable across data sets and judgment tasks [41,42]. Second, it is unclear what model for random agreement among judges is most reasonable for our task. For instance, a uniform distribution for random fragmentation will give an almost zero random agreement between judges and reduce Kappa to percentage agreement. We thus directly analyzed the agreement among annotators along the five dimensions of annotation that we have defined above (*focus*, *polarity*, *certainty*, *evidence *and *trend*).

While the annotation agreement for each sentence was calculated along each of these five dimensions, in order to accurately assess agreement between two annotators on a given sentence, the two had to first produce the same number of fragments for that sentence. Then they were counted as agreeing on a given dimension for that sentence if they assigned the same list of tags in the same order. Thus, the fragments that the two annotators were working with did not have to be identical, but we assumed that if they assigned the same tags in the same order they were detecting the same information in the fragments. This assumption, while not formally validated, was typically satisfied in our many annotation experiments using the changing guidelines throughout the year.

There are two possible ways to handle a comparison of annotations that do not contain the same number of fragments. First, one can score such a comparison as a zero match along all five coordinates. While this is a harsh standard, it has the advantage of not over rating agreement between annotators because a disagreement in number of fragments is an indicator of some level of disagreement regarding the relevant characteristics of the text being annotated. As a second alternative, one can simply exclude from the analysis examples where the fragment numbers disagree. One would do this because in cases where annotators disagree on the number of fragments they may still substantially agree on the characteristics of the text. Further, the annotations may be equally valuable for the eventual goal of learning how to annotate text. We have followed this more optimistic approach for the data reported in Table 2, but adhered to the harsh standard for the remainder of the data, reported in Tables 3, 4, 5.

The data in Table 2 shows the pairwise inter-annotator agreement among the authors. They clearly demonstrate a high level of agreement on the number of fragments. We note that the level of disagreement on fragmentation number is important for the interpretation of the level of agreement on the five dimensions. If there is disagreement on the number of fragments, that sentence is excluded from the remainder of the analysis in Table 2. Just for the data in Table 2 we have used this method of dealing with disagreement in fragment number because we believe it gives a more accurate representation of the true level of agreement between annotators.

Next we compared the performance of the author group (Aut1–3) with that of the group of nine other annotators (Oth1–9). That is, whenever there is an agreed upon majority annotation among the three authors, we compared that annotation with the majority annotation among the Oth1–9 group. The results shown in Table 3 confirm the conclusion already evident from Table 2, that annotations are reproducible at a high level, even among annotators who have had only a brief experience with the guidelines.

To better understand the performance of individual annotators we performed two additional comparisons, both based on the same five dimensions as explained earlier.

A) To check how well the untrained annotators, Oth1–9, performed with respect to the trained annotators, Aut1–3, we scored the annotations from Oth1–9 based on their agreement with those of Auth1–3. That is, for each sentence for which a fragment annotation produced by OthX exactly matched the annotation produced by any one of us (along a certain dimension), OthX received 1 point. A mismatch was assigned 0 points. This way, each annotator could score between 0 and 5 for each sentence, depending on the level of agreement with any one of us. We averaged the results over all 101 sentences. The results of this comparison are shown in Table 4.

B) We next compared the performance of each annotator against the majority obtained over all of Aut1–3 and Oth1–9 together. Each annotator scored a point for an annotation along each dimension if it agreed with the majority. Thus again the score ranged from 0 to 5 points per sentence. We averaged again over the 101 sentences. The results are shown in Table 5.

Clearly there is a significant difference in performance among different annotators. Table 4 shows that five of the Other (Oth) annotators (3, 4, 7, 8 and 9) scored at a level of approximately 3.5 and above. The results in Table 5 allow a more direct comparison of the performance of Aut1–3 and Oth1–9. Authors show the highest agreement with the majority, which is expected given their high level of training in the task. These results also show that all but four of the annotators Oth1–9 perform almost as well as the authors on the annotation task. Based on these results it is expected that a simple use of the guidelines, even without additional instruction, can lead to consistent annotation, as measured by inter-annotator agreement, in about 50% of cases. We of course don't view this as the ultimate desired performance, and additional training is provided to ensure a consistently high level of annotation agreement throughout the data set.

## Discussion

It is challenging to find a non-trivial and useful annotation task that a human can perform and a machine can learn from human-generated data. We believe that we have identified five dimensions of human judgment tasks that are machine-learnable and do have practical implications. Our belief that the tasks are machine-learnable is based on the relatively high level of agreement among untrained annotators, (as shown in Tables 4, 5), who used only the guidelines. Annotation tasks can vary significantly in terms of difficulty: for example, a survey by Saracevic [43] indicated that agreement among human judges varied between 40% and 75% for different tasks. Our results of inter-annotator agreement of 70–80% (see Tables 4 &5) indicate that our annotation problem is relatively easy for the human annotators, which we expect to translate into learnability by machine learning algorithms. Additional support for learnability comes from the observation that clues as to ratings on any of the five dimensions often come in the form of specific words or phrases that occur in the annotated text. This is similar to the sentiment analysis task [37-40] where machine learning has given good results [44]. That said, sentiment analysis work [45,46] also suggests that learning performance depends on topic, domain, and temporality. Thus, conclusions from our work, in which we use biomedical text as a training set, will likely be limited to the sublanguage of biomedicine and not equally applicable to scientific text as a whole.

The variability in annotation agreement along the different dimensions, as summarized in Tables 2 and 3, suggests that categorization along these dimensions is not all of the same difficulty. We were surprised to find that rating of evidence is among the most challenging tasks. While identifying citations (evidence level 2) is an almost mechanical task, there are many subtle ways in which words are used to indicate that a result is a consequence of the new research being reported in a paper (evidence level 3). Similarly, there are many ways to support a statement by eluding to previous work with no specific citation (evidence level 1). Analogous remarks apply to the rating of certainty. For the distinction between *Methodology *versus *Science *or *General *subject matter, we expect a limited set of clue words and phrases to be useful. We expect that distinction between General subject matter versus Science or Methodology may be the most challenging of all the tasks because of the open-ended nature of General subject matter. On a positive note, General subject matter is less common in scientific research articles. While we may therefore expect fewer training examples of General focus, it may make success on this one sub-task less critical to our overall project.

Some insight can be gained from our data about training of annotators. Obviously, a good understanding of the English language and experience in reading scientific literature are important for performing the annotation task as prescribed in our guidelines. It is surprising that even with these skills, (arguably possessed by all 12 annotators), some annotators still performed poorly, as illustrated in Tables 4 and 5. These results strongly indicate that careful quality control is essential, and that poor performance calls for feedback, instruction, and retesting with either resolution of the difficulty or discontinuance from the task. To support such measures, we plan to first train the judges, and then have each sentence annotated independently by three different judges, as well as having different triples of judges assigned to different sentences.

## Conclusion

We have presented guidelines for the annotation of text that have sufficient generality to transcend the confines of a narrow subject area, while supporting practical mining of text for factual information. We have identified five qualitative dimensions that we believe are useful in this respect: *focus*, *polarity*, *certainty*, *evidence*, and *directionality*. We define these dimensions and describe the guidelines we have developed for annotating text with regard to them. Our initial work investigating the reliability of such annotations supports the feasibility of the approach.

Our ultimate goal is the annotation of 10,000 sentences, taken from diverse sources in the biomedical research literature. We believe that with triplicate annotations this will allow the training of machine learning algorithms to perform the annotation task at a useful level of accuracy. Both the annotation and the training of machine learning algorithms are currently ongoing. Should they prove successful, we foresee several areas of application. First, annotation of a large volume of literature and characterization of the literature along the dimensions proposed. This may shed light on the composition of different parts of research papers and even define the characteristics of different genres of biomedical research literature. Another potential application is to combine these annotations with semantic analysis of text to produce a text-mining tool. For example, our annotations could guide entity recognition applied to subject-verb-object triples towards statements that are likely to be highly reliable, as they are supported by evidence or stated in the affirmative with high confidence. Such techniques might also prove helpful to a question answering system and even to a document retrieval system. The scientific literature is vast and there is a wide variety of potential reasons for accessing it. One investigator may wish to obtain validated facts about a particular gene, thus looking for statements of high *Certainty *about it. A second investigator may desire to examine contradicting statements regarding the expression of a gene; in this case statements mentioning the same gene but with opposite *Polarity *and/or opposite *Direction/Trend *are important. A third investigator may wish to examine uncertain hypotheses regarding this same gene, which would involve looking for statements with a low *Certainty *level. Such statements may stimulate his thinking and lead him in new research directions. In fact we suggest that contradictions and speculations in the literature are likely to prove a fruitful source of new hypotheses. All of this is territory yet to be explored.

## Methods

The annotation guidelines presented here evolved through several iterations over a period of more than a year. We repeatedly tested and revised the guidelines by independently annotating text ranging in style from reviews to research publications, from several biomedical domains, and comparing our results. This resulted in the guidelines in their current form, along with appendices containing numerous examples illustrating the principles laid out in the guidelines.

Our general aim is to identify information-bearing fragments within scientific text, in order to substantiate our knowledge about important biomedical entities and processes. Furthermore, we would like to differentiate these informative fragments from non-informative ones automatically, as well as to distinguish among several types of informative fragments. To simplify the task we typically annotate at the sentence level where possible, but complex sentences are annotated as needed at the level of subsentential fragments. Such fragmentation is necessary to capture changes in focus, polarity, certainty, evidence, or trend that may, and frequently do, occur within a single sentence. For example the sentence: "*Furthermore, Bax insertion into the MOM of Myc-/- cells appeared to be efficient, which would not be expected for a fall-back pathway.*" exhibits a change in polarity in the final clause. It would be necessary to fragment it at this point in order to capture the fact that two different polarities exist in the sentence. The only rule for fragmentation is that it occurs only when there is a change in value along any of the five annotation dimensions.

We are currently pursuing two related sub-goals: 1) to manually annotate a sizable corpus, and 2) to use this corpus to build and train text-classifiers. To approach the first subtask, annotation of a biomedical corpus, we characterize text fragments along the following dimensions:

### Focus

Each text fragment may convey one (and sometimes more) of:

• **Scientific **content, findings and discovery; we refer to this type of information as *Science*, and indicate it by the tag **S**.

• **Generic-level **information; General state of knowledge and science outside the scope of the paper, the structure of the paper itself or the state of the world. Such statements are not usually based on scientific experiment, and may reflect an opinion or an observation that would have been as truthful, and probably as valid, if made by a layperson. We refer to it as *Generic*, and denote it with the tag **G**.

• **Methodology **that was used in an experiment or a study. We refer to it as *Methodology*, and denote it with the tag **M**.

We note that the focus of a statement may be viewed differently depending on the context (e.g. section, paragraph, sentence) in which it appears. What may be regarded as a scientific finding in one context is a methodology in another. In fact, most scientific methods are based on what were at one time reported scientific findings. Thus the annotator will inevitably face ambiguity in trying to distinguish science and methodology. Our approach is therefore only to annotate methodology when the sentence under annotation contains an indication that methodology is being discussed. In contrast to zone-based annotation schemes, we note that not every sentence appearing in a Methodology section discusses methodology, and not every sentence discussing methodology appears in the Methodology section. Further, nothing is gained if we annotate a sentence as methodology when it is indistinguishable from sentences discussing science. We are interested in learning how the text of a sentence itself signals that methodology is being discussed. See Appendices B–F for annotated examples [47].

### Polarity

A fragment with any focus can be stated either positively (**P**) or negatively (**N**). For statements that convey lack-of-knowledge, (e.g. "It is still unknown whether..."), the default assignment is **P**. The lack of knowledge in this case will be reflected by a *certainty *degree of 0, as explained in the next item. Every fragment should be annotated by its polarity, regardless of its focus or its certainty.

### Certainty

Each fragment conveys a degree of certainty about the validity of the assertion it makes. Our annotation uses a scale in the range *0–3 *as a measure of certainty, for both *positive *and *negative *statements. The lowest degree (**0**) represents *complete uncertainty*, that is, the fragment explicitly states that there is an uncertainty or lack of knowledge about a particular phenomenon ("*it is unknown...*" or "*it is unclear whether...*" etc.). The highest degree, (**3**), represents complete certainty, reflecting an accepted, known and/or proven fact. The intermediate degree (**1**) represents a low certainty, while (**2**) is assigned to high-likelihood expressions that are still short of complete certainty.

### Evidence

This dimension indicates for any fragment, regardless of its focus and certainty, if its assertion is supported by evidence. The existence – or the lack – of evidence is denoted by a tag starting with the letter **E**. The letter is followed by one or more digits, in the range *0–3*, indicating the type of evidence or its absence:

• **E0**: No indication of evidence in the fragment whatsoever, or an explicit statement in the text indicates *lack of evidence*.

• **E1**: A claim of evidence, but no verifying information is explicitly given. Evidence is not shown within the annotated sentence/fragment, and no explicit reference to it is provided. The evidence is merely asserted to exist in some form, possibly in the preceding text, or in prior experiments, but its location is not explicitly stated. Note that in this case the indirect implication of evidence may not be explicit in the fragment, but implied by a use of terms referring to a previous fragment. For instance, a sentence may begin with the fragment "*Previous experiments show that...*", followed by the fragment, "*therefore, it is likely that ...*". Both fragments are of evidence level 1; the first because it points to experiments without an explicit reference, and the second, because of the "*therefore*" term which uses the previous assertion as an indirect evidence.

• **E2**: Evidence is not given within the sentence/fragment, but *explicit *reference is made to other *papers *(citations) to support the assertion.

• **E3**: Evidence is provided, within the fragment, in one of the following forms:

○ A reference to experiments previously reported within the body of the paper by a direct description of the finding as an experimental result (e.g. "Our data indicates...", "...our results show"...)

○ A verb (typically in the past-tense) within the statement indicates an observation or an experimental finding which is described within the paper, (e.g. "We found that...", "We see that...").

○ A reference to an experimental figure or a table of data given within the paper.

A statement about a certain finding may be assigned different levels of evidence depending on the wording used. For instance, something reported as a finding by the authors would be annotated as *E3*. (e.g., "*Our data demonstrate that ICG-001 has no effect on AP1 ...*"). In this case the words "*Our data demonstrate*" indicate the evidence. However, a similar statement may occur without any indication of evidence. (e.g., "*ICG-001 has no effect on AP1 ...*"). In that case, stated without any support, it would be annotated as *E0*. This same statement would be annotated as *E1 *if accompanied by a non-explicit reference (e.g. "*Previous studies suggest that ICG-001 has no effect on AP1 ...*"). Finally, if explicit reference to the original work is given: "*Previous studies suggest that ICG-001 has no effect on AP1 ... [25]*", the tag would be *E2*.

We note that it is not the scientific details themselves, be they ever so intricate, that constitute the evidence. Rather, it is the specific wording that points to a certain type of evidence.

### Direction/trend

The signs + **or **- indicate respectively whether the assertion reports a qualitatively *high *or *low *level or an *increase*/*decrease *in a specific phenomenon, finding or activity.

This tag is introduced to separate the notion of positive/negative results and assertions (as captured by *Polarity*) from the level of the observed phenomenon itself. For instance, the sentence: "*In fact, as demonstrated using several SOD assays including pulse radiolysis, 2-ME does not inhibit SOD*" indicates a negative experimental finding ("*does not...*", negative polarity), about a negative trend ("*inhibit*"). This is a case known as *double-negation*, and is typically hard to annotate, as it is not clear whether the phenomenon is actually present or not. Separating *Direction *from *Polarity *provides a mechanical way to annotate and interpret such statements. Moreover, this separation also provides the means to indicate presence/absence of experimental findings, annotated using *Polarity*, regardless of whether these findings demonstrate the presence or the absence of the monitored phenomenon, as that latter is captured by the *Trend*.

The guidelines in full detail as well as numerous annotated examples are publicly available at [47,48].

## Authors' contributions

All three authors shared equally in developing the guidelines presented here. HS compiled the guidelines into written form. AR obtained the judgments from the nine non-authors who participated in the study. WJW performed the data analysis. All authors read and approved the final manuscript.

## Figures and Tables

**Table 1:** Annotation characteristics. Each column represents an annotator: Aut1–3 are the authors of this paper and Oth1–9 are the other annotators. The first five rows show the number of fragments into which sentences were broken by each of the annotators. The counts appearing in subsequent rows are normalized by the number of fragments created by that annotator.

		Aut1	Aut2	Aut3	Oth1	Oth2	Oth3	Oth4	Oth5	Oth6	Oth7	Oth8	Oth9
Number of fragments	*1*	88	80	86	40	60	85	81	40	47	81	68	83
	*2*	10	18	12	42	31	16	17	44	44	19	30	16
	*3*	2	3	3	18	7	0	3	13	8	1	3	2
	*4*	1	0	0	1	2	0	0	4	2	0	0	0
	*5*	0	0	0	0	1	0	0	0	0	0	0	0
Focus	*G*	0.09	0.15	0.13	0.27	0.29	0.16	0.22	0.15	0.2	0.08	0.13	0.07
	*M*	0.24	0.15	0.23	0.15	0.15	0.18	0.18	0.09	0.15	0.36	0.23	0.15
	*S*	0.71	0.8	0.82	0.63	0.63	0.8	0.63	0.75	0.65	0.58	0.71	0.79
Polarity	*P*	0.94	0.94	0.93	0.81	0.92	0.92	0.92	0.88	0.81	0.94	0.92	0.83
	*N*	0.06	0.06	0.07	0.19	0.08	0.08	0.08	0.12	0.19	0.05	0.08	0.17
Certainty	*0*	0.02	0.02	0.04	0.01	0	0.02	0.03	0.04	0.01	0.03	0.01	0.08
	*1*	0.07	0.05	0.13	0.04	0.1	0.05	0.07	0.02	0.04	0.11	0.02	0.03
	*2*	0.11	0.10	0.13	0.04	0.22	0.05	0.10	0.01	0.05	0.18	0.12	0.03
	*3*	0.81	0.83	0.69	0.91	0.68	0.88	0.81	0.92	0.9	0.68	0.86	0.87
Evidence	*0*	0.31	0.34	0.26	0.75	0.38	0.27	0.27	0.7	0.83	0.07	0.31	0.42
	*1*	0.11	0.09	0.09	0.03	0.07	0.09	0.14	0.03	0	0.43	0.15	0.10
	*2*	0.19	0.21	0.24	0.14	0.11	0.23	0.23	0.2	0.13	0.16	0.3	0.31
	*3*	0.41	0.36	0.43	0.08	0.44	0.4	0.4	0.07	0.05	0.34	0.28	0.19
Trend	+	0.13	0.03	0.13	0.12	0.12	0.03	0.07	0.03	0.10	0.05	0.11	0.03
	-	0.19	0.06	0.17	0.19	0.12	0.07	0.13	0.04	0.16	0.06	0.08	0.11
	*0*	0.68	0.91	0.70	0.69	0.76	0.90	0.80	0.93	0.74	0.89	0.81	0.86

**Table 2:** Pairwise agreement among the authors. The numbers presented in rows 3–7 were calculated for each pair of authors, as: (# of annotation agreements)/(# of fragments mutually annotated) but only included those sentences on which the pair of authors agreed on the number of fragments (such agreement on fragment number as a percentage is given in row 2).

Dimension	Aut1–Aut2	Aut2–Aut3	Aut1–Aut3
# of fragments	0.851	0.881	0.891
Focus	0.803	0.731	0.733
Polarity	1.000	0.989	0.989
Certainty	0.861	0.787	0.800
Evidence	0.757	0.787	0.844
Trend	0.803	0.775	0.866

**Table 3:** Comparison of the majority annotation among Aut1–3, with the majority annotation from Oth1–9. As the numbers total to the 101 sentences in each row, these numbers can essentially be interpreted as percentage.

Dimension	No Aut1–3 Majority	Aut1–3 agrees with Oth1–9 Majority	Aut1–3 disagrees with Oth1–9 Majority
# of fragments	2	88	11
Focus	15	73	13
Polarity	2	86	13
Certainty	7	77	17
Evidence	6	79	16
Trend	9	76	16

**Table 4:** Individual scores of Oth1–9 compared with Aut1–3. Average sentence scores based on 1 point awarded for each agreement with any one of Aut1–3. The maximum possible score is 5, while 0 is the minimum.

Annotator	Oth1	Oth2	Oth3	Oth4	Oth5	Oth6	Oth7	Oth8	Oth9
Average Score	2.10	2.96	4.25	4.22	2.15	2.53	3.84	3.62	3.84

**Table 5:** Individual scores for all annotators. Average sentence scores with 1 point awarded for each agreement with the majority annotation determined over the whole set of twelve annotators. The maximum possible score is 5, while 0 is the minimum.

Annotator	Aut1	Aut2	Aut3	Oth1	Oth2	Oth3	Oth4	Oth5	Oth6	Oth7	Oth8	Oth9
Ave. Score	3.96	4.25	4.08	2.06	2.95	4.00	4.00	2.07	2.50	3.59	3.59	3.57
